# Genetic dissection of heterosis of *indica*–*japonica* by introgression line, recombinant inbred line and their testcross populations

**DOI:** 10.1038/s41598-021-89691-6

**Published:** 2021-05-13

**Authors:** Wenqing Yang, Fan Zhang, Sundus Zafar, Junmin Wang, Huajin Lu, Shahzad Naveed, Jue Lou, Jianlong Xu

**Affiliations:** 1 Southern Zhejiang Key Laboratory of Crop Breeding, Wenzhou Vocational College of Science and Technology, Wenzhou, 325006 Zhejiang China; 2 Institute of Crop Sciences/National Key Facility for Crop Gene Resources and Genetic Improvement, Chinese Academy of Agricultural Sciences, Beijing, 100081 China; 3 Shenzhen Branch, Guangdong Laboratory for Lingnan Modern Agriculture, Agricultural Genomics Institute at Shenzhen, Chinese Academy of Agricultural Sciences, Shenzhen, 518120 China; 4 The Institute of Crops and Nuclear Technology Utilization, Zhejiang Academy of Agricultural Sciences, Hangzhou, 310021 Zhejiang China

**Keywords:** Genetics, Agricultural genetics, Genetic linkage study

## Abstract

The successful implementation of heterosis in rice has significantly enhanced rice productivity, but the genetic basis of heterosis in rice remains unclear. To understand the genetic basis of heterosis in rice, main-effect and epistatic quantitative trait loci (QTLs) associated with heterosis for grain yield-related traits in the four related rice mapping populations derived from Xiushui09 (XS09) (*japonica*) and IR2061 (*indica*), were dissected using single nucleotide polymorphism bin maps and replicated phenotyping experiments under two locations. Most mid-parent heterosis of testcross F_1_s (TCF_1_s) of XS09 background introgression lines (XSILs) with Peiai64S were significantly higher than those of TCF_1_s of recombinant inbred lines (RILs) with PA64S at two locations, suggesting that the effects of heterosis was influenced by the proportion of introgression of IR2061’s genome into XS09 background. A total of 81 main-effect QTLs (M-QTLs) and 41 epistatic QTLs were identified for the phenotypic variations of four traits of RILs and XSILs, TCF_1_s and absolute mid-parent heterosis in two locations. Furthermore, overdominance and underdominance were detected to play predominant effects on most traits in this study, suggesting overdominance and underdominance as well as epistasis are the main genetic bases of heterosis in rice. Some M-QTLs exhibiting positive overdominance effects such as *qPN1.2*, *qPN1.5* and *qPN4.3* for increased panicle number per plant, *qGYP9* and *qGYP12.1* for increased grain yield per plant, and *qTGW3.4* and *qTGW8.2* for enhanced 1000-grain weight would be highly valuable for breeding to enhance grain yield of hybrid rice by marker-assisted selection.

## Introduction

Food security is one of the major concerns of the rapidly growing population of the world, which can be solely resolved through the sustainable food production. In particular, rice is a staple food for more than half of the population globally^1^, which is usually classified into two main types, *indica* and *japonica*. These two subspecies considerably differ from each other on the basis of morphology and physiology, while inter-subspecies hybrid usually demonstrated a strong level of heterosis for numerous agronomic traits^2^.

Heterosis, also known as hybrid vigor, plays a key role in the enhancement of crop yield and thus, regarded as a far superior phenotypic performance of hybrids in comparison with their parents^3^. F_1_-hybrid plants possess appreciable features such as increased yield, biomass, vegetative growth rate, and tolerance of biotic and abiotic stresses^4^. The much enhanced yield of F_1_-hybrid has been utilized in numerous crops and vegetables^5^. Jones has firstly reported the heterosis in rice and suggested that few F_1_-hybrid plants had more culms and higher yield as compared with their parents^6^. Later, numerous researchers have reported heterosis for yield and yield-related traits^7–10^. Notably, China had successfully achieved the application of heterosis of rice into agronomic industries, and the planted land of hybrid rice ever rose to as high as 57% of the total rice area in China^11^. However, the traditional heterosis cannot satisfy the breeding demands of improved yield^12^.

Several researchers revealed that the *indica*–*japonica* hybrids have large sink and genetic source, capable of increasing the yield potential. As a result, many studies investigated the potential of *indica*–*japonica* hybrids^13–15^. However, hybrid sterility seriously restricts the development of high-yield hybrid rice^15^. Thus, the development of *indica*–*japonica* background lines is prerequisite for high yielding hybrid^16^. Numerous inter-subspecific lines have been developed for hybrid varieties with stronger heterosis and normal seed set^17–19^. Recently, China has made a great success in utilization of heterosis of *indica*–*japonica*, releasing many super hybrid combinations such as Yongyou 6, Yongyou 9, Yongyou 12, Chunyou658, Chunyou84, Chunyou 927 by introgressing *japonica* composition and the wide-compatibility gene (*S5*^*n*^) into *indica* restorer line background^20,21^. In spite of the extensive research, the genetic basis of heterosis of *indica*–*japonica* are not yet completely understood.

With the advancement of second-generation genome sequencing technologies, genome-wide association study (GWAS) has been acquired to identify quantitative trait loci (QTLs) for rice agronomic traits^22,23^. GWAS provided insights into the genetic architecture of the heterosis for yield traits in rice^24,25^, and permitted the rapid identification of single nucleotide polymorphisms (SNPs) associated with heterosis. Based on QTL analysis, dominance^26^, overdominance^17,27,28^ and epistasis^29,30^ could be the key determinants of heterosis in rice^31^. Testcrossing is one the most regular method to identify superior hybrid in plant breeding. Therefore, testcross populations have been extensively used to identify QTLs associated with yield heterosis in rice, suggesting that these QTL effects may sufficiently describe the genetic basis of heterosis^32,33^. While, the high perseverance of genetic and field data was required to detect significant QTLs in the rice genome associated with hybrid performance^34–36^. However, the reports demonstrated the SNPs analysis of heterosis in *indica*–*japonica* background lines is surprisingly rare. By using 1,654,030 SNPs of 1,495 hybrids and their parental lines of rice, Li et al*.* reported the genetic basis of heterosis and the involvement of superior allele to heterosis^37^. Meanwhile, Zhen et al*.* performed GWAS using 50 K SNP for F_1_-hybrid and identified many QTLs related to yield traits, and genes including *Hd3a*, *qGL3* and *LAX2* within these QTLs were detected^25^.

In this study, we performed genotyping using a customized rice 56 K SNP array to Xiushui09 (XS09) background introgression lines (XSILs) and recombinant inbred lines (RILs) derived from *indica–japonica* varieties and their testcross F_1_ (TCF_1_) populations separately, and identified 81 QTLs underlying heterosis for four yield traits with a number of already identified genes within these QTLs. We further investigated divergence and the gene actions of QTLs responsible for yield in the inbred line populations and heterosis in the testcross populations to understand the relative significance of additive and non-additive gene actions in rice improvement.

## Results

### Phenotypic performances

The performance for the four yield traits of the parents (XS09 and IR2061) of the RILs and XSILs, the tester line Peiai64S (PA64S) (or PA64), the relative F_1_ plants (XS09 × IR2061 and PA64S × XS09), and theirs mid-parent heterosis (*H*_MP_) in Lingshui (LS) and Wenzhou (WZ) were shown in Table 1. Significant differences were observed for filled grain number per panicle (FGNP) and 1000-grain weight (TGW) between XS09 and IR2061 in both locations. The F_1_ (XS09 × IR2061) plants had significantly higher panicle number per plant (PN) and FGNP, and similar TGW and grain yield per plant (GYP) compared with the two parents in the two locations. The *H*_MP_ of the F_1_ (XS09 × IR2061) plants for PN, FGNP and GYP was 25.9%, 37.2%, and − 0.6% in LS, and 41.0%, 37.7%, and 3.6% in WZ, respectively, showing strong heterosis for PN and FGNP between the two parents and low heterosis for GYP with low fertility of F_1_ plants in the two locations, resulting from incompatibility between the two subspecies parents. As compared with XS09, the tester line PA64S had significantly higher PN, FGNP, and GYP, and significantly lower TGW in LS. In the WZ experiment, PA64 (the recurrent parent of PA64S) was used instead of PA64S due to its sterility, which showed significantly higher PN, lower FGNP and TGW than XS09, and similar GYP with XS09. The F_1_ (PA64S × XS09) plants showed significantly increased FGNP and GYP, and similar PN compared with the better parent PA64S/PA64 in both locations, and similar TGW in LS and significantly lower TGW in WZ compared with the better parent XS09. Significant *H*_MP_ of the F_1_ (PA64S × XS09) plants were found with 27.0% for PN, 59.0% for FGNP and 60.7% for GYP in LS, and 27.7% for PN, 94.8% for FGNP and 53.3% for GYP in WZ.

**Table 1:** Phenotypic performance and mid-parent heterosis of four yield traits in the parent lines, RIL and XSIL populations from a cross of Xiushui09/IR2061, and their testcross F_1_s with common maternal tester line PA64S in Lingshui (LS) and Wenzhou (WZ).

Location	Type^a^	No. of samples	PN	FGNP	TGW (g)	GYP (g)
LS	XS09	1	10.4 ± 0.6c	95.9 ± 5.2d	24.5 ± 0.2a	24.6 ± 2.7c
	IR2061	1	11.1 ± 0.5c	130.5 ± 8.4c	22.4 ± 0.3b	27.5 ± 3.5c
	PA64S	1	16.1 ± 0.1a	146.2 ± 5.4bc	20.0 ± 0.2c	37.2 ± 1.7b
	XS09 × IR2061 F_1_	1	13.5 ± 0.8b	155.3 ± 11.0b	23.1 ± 0.4ab	25.9 ± 3.2c
	XS09 × IR2061 *H*_*MP*_ (%)	–	25.9*	37.2*	− 1.8	− 0.6
	PA64S × XS09 F_1_	1	16.8 ± 0.8a	192.4 ± 6.2a	24.3 ± 1.1a	49.6 ± 4.1a
	PA64S × XS09 *H*_*MP*_ (%)	–	27.0*	59.0**	9.2	60.7*
	RILs	209	11.4 ± 3.0	140.3 ± 38.7	27.3 ± 3.5	28.9 ± 6.9
	RILTCF_1_s	209	16.7 ± 3.1	192.5 ± 30.1	26.0 ± 1.9	53.1 ± 8.9
	RILTCF_1_s *H*_*MP*_ (%)	–	21.5 ± 18.3	35.5 ± 20.0	9.8 ± 4.6	61.6 ± 27.9
	XSILs	222	12.1 ± 1.8	124.8 ± 25.5	25.9 ± 2.1	25.3 ± 5.1
	XSILTCF_1_s	222	18.4 ± 2.0	198.2 ± 20.1	24.0 ± 1.0	52.5 ± 6.4
	XSILTCF_1_s *H*_*MP*_ (%)	–	30.6 ± 12.6	47.0 ± 15.8	4.7 ± 3.0	69.1 ± 23.3
WZ	XS09	1	8.3 ± 0.3c	124.4 ± 5.9c	23.9 ± 0.6a	26.5 ± 2.4b
	IR2061	1	9.5 ± 0.4c	89.8 ± 4.3d	20.7 ± 0.6b	22.9 ± 1.1b
	PA64	1	14.1 ± 0.8ab	68.5 ± 8.8d	20.7 ± 0.3b	26.4 ± 1.3b
	XS09 × IR2061 F_1_	1	12.6 ± 0.5b	147.5 ± 8.6b	22.5 ± 0.5ab	25.6 ± 4.0b
	XS09 × IR2061 *H*_*MP*_ (%)	–	41.0**	37.7*	0.8	3.6
	PA64S × XS09 F_1_	1	14.3 ± 0.8a	187.8 ± 11.9a	21.5 ± 1.3b	40.5 ± 3.7a
	PA64S × XS09 *H*_*MP*_ (%)	–	27.7*	94.8**	− 3.7	53.3*
	RILs	209	12.1 ± 2.9	112.8 ± 30.6	21.9 ± 3.6	18.9 ± 6.7
	RILTCF_1_s	209	16.1 ± 2.4	154.6 ± 29.2	21.4 ± 2.1	40.5 ± 7.7
	RILTCF_1_s *H*_*MP*_ (%)	–	23.5 ± 16.9	74.5 ± 41.6	1.0 ± 10.1	83.0 ± 44.9
	XSILs	222	11.0 ± 2.0	116.5 ± 20.9	17.2 ± 3.1	15.8 ± 4.8
	XSILTCF_1_s	222	17.1 ± 2.6	175.1 ± 26.2	18.3 ± 2.2	42.0 ± 8.7
	XSILTCF_1_s *H*_*MP*_ (%)	–	36.3 ± 18.5	91.5 ± 35.6	− 3.4 ± 12.2	102.1 ± 49.5

^a^*PA64S* Peiai64S, *PA64* the recurrent parent of an isogenic to PA64S, *XS09* Xiushui09, *RILs* recombinant inbred lines derived from a cross between Xiushui09 and IR2061, *XSILs* introgression lines under Xiushui09 background with IR2061 as a donor, *TCF*_*1*_*s* testcross F_1_s between RILs or XSILs and one common maternal tester line PA64S, *H*_*MP*_ mid-parent heterosis, *FGNP* filled grain number per panicle; *TGW* 1000-grain weight, *PN* effective panicle number per plant, *GYP* grain yield per plant.

Trait values are presented as mean ± sd.

Characters behind the sd value indicate significant differences based on Duncan’s multiple comparison tests (*P* < 0.05) ‘*’ and ‘**’ indicate significant differences between mean F_1_ hybrid and average performance of corresponding parental lines using Student’s *t*-tests at *P* < 0.05 and *P* < 0.01, respectively.

**Figure 1:**
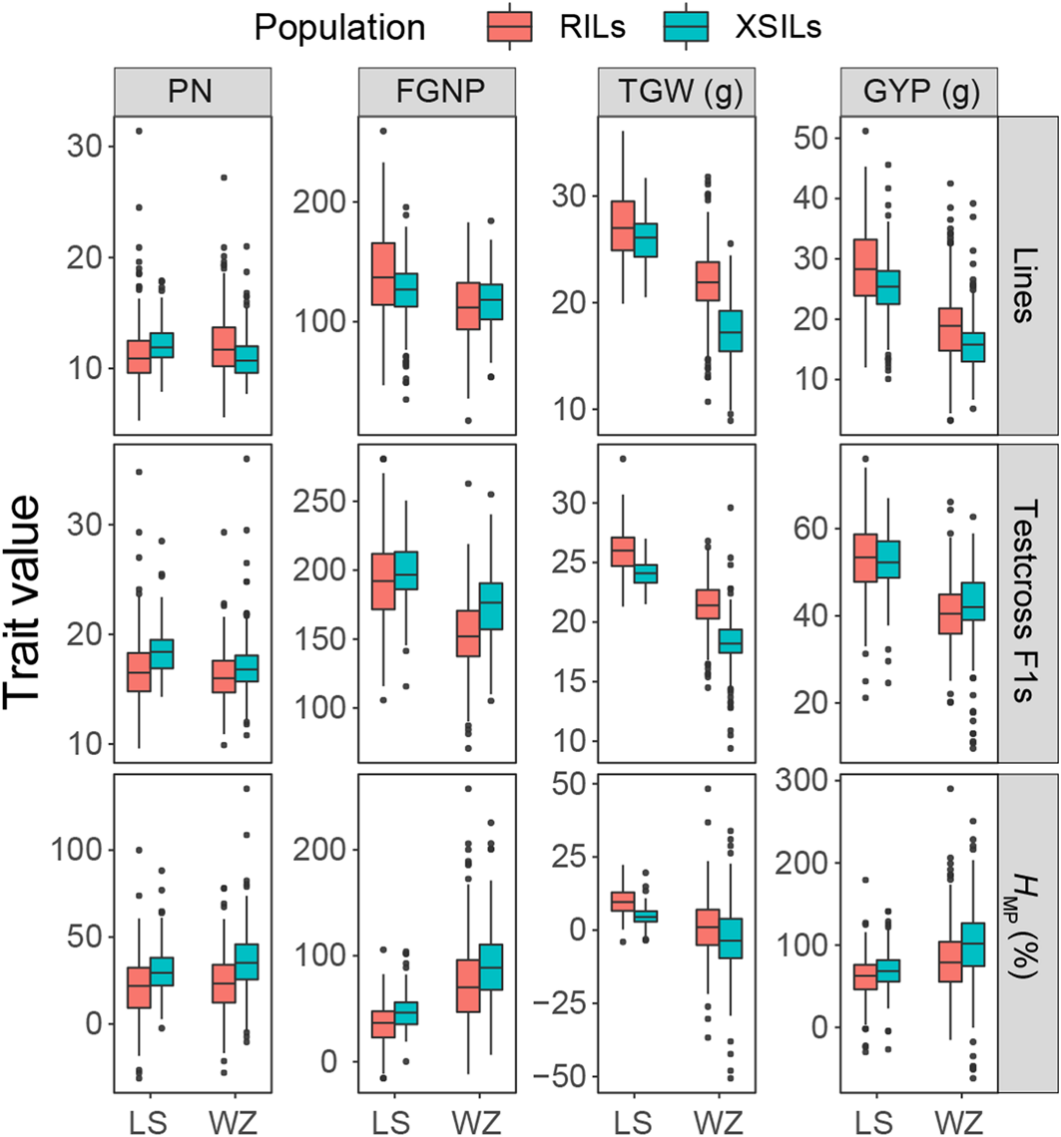
Phenotypic distribution for the four yield-related traits of two populations at the two locations. *RILs* recombinant inbred line derived from a cross between Xiushui09 and IR2061, *XSILs* introgression lines under Xiushui09 background with IR2061 as a donor, *TCF*_*1*_ testcross F_1_ between RIL and XSIL and one common maternal tester line PA64S, *H*_*MP*_ mid-parent heterosis, *LS* Lingshui, *WZ* Wenzhou, *GYP* grain yield per plant, *FGNP* filled grain number per panicle, *TGW* 1000-grain weight, *PN* effective panicle number per plant.

The ranges of variations for each trait in the lines (RILs and XSILs), TCF_1_s (TCF_1_s of RILs with PA64S [RILTCF_1_s] and TCF_1_s of XSILs with PA64S [XSILTCF_1_s]) and their *H*_MP_s were shown in Fig. 1. Wider distributions of all traits were found in the RIL population than XSIL population in both locations. The RILTCF_1_s had significantly lower TGW and higher PN, FGNP and GYP than the RILs at the two locations, while similar results were observed for all traits except for TGW between XSILTCF_1_s and XSILs in WZ (Fig. 1, Table 1). The mean TGW and GYP in RILTCF_1_s and the mean PN, FGNP and GYP in XSILTCF_1_s were all higher than those of the check F_1_ (PA64S × XS09) plants in LS. There were 7 (3.3%), 18 (8.6%), 27 (12.9%), and 8 (3.8%) RILTCF_1_s and 16 (7.2%), 16 (7.2%), 0, and 3 (1.4%) XSILTCF_1_s having significantly higher PN, FGNP, TGW, and GYP than the F_1_ (PA64S × XS09) plants in LS, respectively. Similarly, in the WZ experiment, the mean PN in both RILTCF_1_s and XSILTCF_1_s and the mean GYP in XSILTCF_1_s were higher than the check F_1_ (PA64S × XS09) plants. There were 28 (13.4%), 2 (1.0%), 1 (0.5%), and 1 (0.5%) RILTCF_1_s and 39 (17.6%), 1 (0.5%), 0, and 4 (2.0%) XSILTCF_1_s having significantly higher PN, FGNP, TGW, and GYP than the F_1_ (PA64S × XS09) plants, respectively. On average, the RILTCF_1_s had higher TGW and GYP and lower PN and FGNP than XSILTCF_1_s in LS, while a similar trend was observed in RILTCF_1_s for PN, FGNP and TGW, but lower GYP compared with XSILTCF_1_s in WZ.

The performance of the hybrid plants was determined by their mid-parental value and the heterosis level. Here, the most *H*_MP_ of the TCF_1_s showed positive heterosis in all traits except for TGW in both locations (Fig. 1, Table 1). Moreover, the *H*_MP_ of the TCF_1_s for FGNP and GYP were generally higher than those of PN and TGW. We found that 39.2%, 10.5%, 53.6%, and 54.1% of the RILTCF_1_s and 58.6%, 19.4%, 0, and 64.0% of the XSILTCF_1_s with higher *H*_MP_ of PN, FGNP, TGW and GYP compared with the check F_1_ (PA64S × XS09) plants in LS, respectively. Similarly, 37.3%, 25.8%, 68.9%, and 77.5% of the RILTCF_1_s and 69.4%, 42.8%, 50.9%, and 88.3% of the XSILTCF_1_s with higher *H*_MP_ of PN, FGNP, TGW and GYP compared with the check F_1_ (PA64S × XS09) plants in WZ, respectively. The mean *H*_MP_ of the XSILTCF_1_s was higher for PN, FGNP and GYP but lower for TGW than those of the RILTCF_1_s at the two locations, suggesting that the effects of heterosis may be influenced by the proportion of introgression of IR2061’s genome into the XS09 background, when RILs or XSILs were tested with the common *indica* tested parent PA64S. Taking together, the observed heterosis for GYP in the TCF_1_s was mainly attributed to yield-component traits PN and FGNP.

### Correlations of yield traits among lines, testcross F_1_s and mid-parent heterosis

Correlation analyses on each trait across the lines (RILs or XSILs), the TCF_1_s (RILTCF_1_s and XSILTCF_1_s), and their *H*_MP_s are presented in Table 2. The *H*_MP_s of TCF_1_s were determined by the trait values of the TCF_1_s and the corresponding lines (RILs or XSILs). In other words, the performance of individual TCF_1_ plants largely depends on the cumulative effect of the trait value of the relative line and the heterosis. There were significant and highly positive correlations between the lines and the relative TCF_1_s for all traits in the two locations except FGNP between XSILs and the XSILTCF_1_, and GYP between RILs and the RILTCF_1_ in WZ, and the correlation coefficients in LS were generally higher than those in WZ. The significant and highly positive correlations (*r* ≥ 0.53, *P* < 0.01) between TCF_1_s and their *H*_MP_s were also observed for PN, FGNP and GYP in both locations, while the correlation of TGW was slightly weak between RILTCF_1_s and their *H*_MP_s (*r* = 0.26) and between XSILTCF_1_s and their *H*_MP_s (*r* = 0.15) in LS. In contrast, most correlations between the *H*_MP_ of TCF_1_s and the relative lines for FGNP, TGW and GYP were significantly negative and were much stronger than PN in both locations (Table 2). In addition, the correlations between the lines and their *H*_MP_ for these yield traits differed according to different locations. The above results suggested that the role of heterotic loci for yield should be affected by the environment.

**Table 2:** Correlation coefficients of the four yield traits among inbred line populations, testcross F_1_s, and mid-parent heterosis in Lingshui (LS) and Wenzhou (WZ).

Location	Population	Item	PN	FGNP	TGW	GYP
LS	RIL	Line vs Testcross F_1_	0.57**	0.49**	0.83**	0.26**
		Line vs *H*_*MP*_	− 0.06	− 0.39**	− 0.32**	− 0.34**
		Testcross F_1_ vs *H*_*MP*_	0.78**	0.60**	0.26**	0.81**
	XSIL	Line vs Testcross F_1_	0.43**	0.47**	0.82**	0.16*
		Line vs *H*_*MP*_	− 0.19**	-0.49**	− 0.43**	− 0.47**
		Testcross F_1_ vs *H*_*MP*_	0.80**	0.53**	0.15*	0.79**
WZ	RIL	Line vs Testcross F_1_	0.46**	0.24**	0.45**	0
		Line vs *H*_*MP*_	− 0.28**	− 0.57**	− 0.42**	− 0.59**
		Testcross F_1_ vs *H*_*MP*_	0.71**	0.62**	0.61**	0.78**
	XSIL	Line vs Testcross F_1_	0.38**	0.07	0.29**	− 0.15*
		Line vs *H*_*MP*_	− 0.15*	− 0.58**	− 0.39**	− 0.54**
		Testcross F_1_ vs *H*_*MP*_	0.85**	0.76**	0.77**	0.90**

*RIL* recombinant inbred line derived from a cross between Xiushui09 and IR2061, *XSIL* introgression lines under Xiushui09 background with IR2061 as a donor, *H*_*MP*_ mid-parent heterosis, *PN* effective panicle number per plant, *FGNP* filled grain number per panicle; *TGW* 1000-grain weight, *GYP* grain yield per plant.

‘*’ and ‘**’ indicate significant correlation at *P* < 0.05 and *P* < 0.01, respectively.

### Effects of proportion of *Japonica* genome in male lines on heterosis of testcross

**Figure 2:**
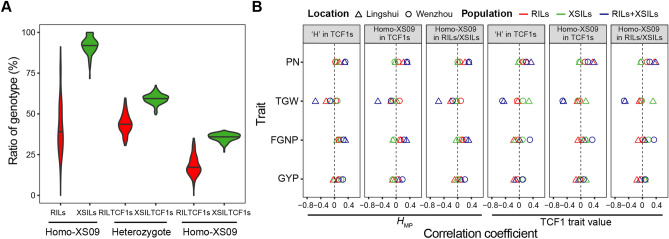
Relationship between the ratio of heterozygous and homozygous *japonica* genotype and the heterosis in rice. (**A**) Distribution of the ratio of heterozygote and homozygous XS09 genotypes in RIL and XSIL populations and testcross F_1_s. (**B**) Correlations between the ratio of the heterozygous and homozygous genotype of rice individuals and the value of relative TCF_1_s and mid-parent heterosis. ‘Homo’ and ‘H’ indicates homozygote and heterozygote, respectively.

PA64S shared the same genotypes with XS09 at 9646 (38.1%) of all 25,296 SNPs. Within the RIL population, individual RILs varied considerably in their ratios of homozygous XS09 alleles ranging from 0 to 91% with a median of 42%, much smaller than the ratios of homozygous XS09 alleles in the XSILs ranging from 72 to 100% with a median of 92% due to two-time consecutive backcrossing (Fig. 2A). Furthermore, most XSILTCF_1_s had a higher ratio of homozygous XS09 alleles (a median of 36% with a range from 27 to 40%) and a higher ratio of heterozygous genotype (a median of 60% with a range from 50 to 68%) than RILTCF_1_s. In view of a continuous distribution of *japonica* genome (XS09 genome) in the two inbred line (XSILs and RILs) populations, the two populations could be also merged into one combined population for further analysis of the proportional effect of *japonica* genome on heterosis of the combined testcross populations. Thus, at all tested SNPs, the ratio of *japonica* genome (XS09-genome) could range from 0 to 100% in the combined inbred population consisted of all RILs and XSILs, and from 0 to 40% in a combined TCF_1_ population consisted of RILTCF_1_s and XSILTCF_1_s. Correlation analyses between the ratios of XS09 genome of individual inbred line and the trait value or *H*_MP_ of the testcross for the four yield traits were shown in Fig. 2B and Table S1. The results indicated that there were significant negative correlations for TGW, significant weak positive correlations for PN and FGNP and no significant correlations for GYP between the trait values or *H*_MP_ of the TCF_1_s or the combined TCF_1_s and the ratios of XS09 genome in the RILs, XSILs and the combined line population (RILs and XSILs). It should be noted that these significant correlations found in the combined line population were much stronger than those only in the RILs or XSILs. It was indicated that there was no inevitable connection between heterosis of the testcross and the proportion of *japonica* genome in paternal lines in our tested populations.

### Main-effect QTL mapping of yield-related traits and mid-parent heterosis

**Figure 3:**
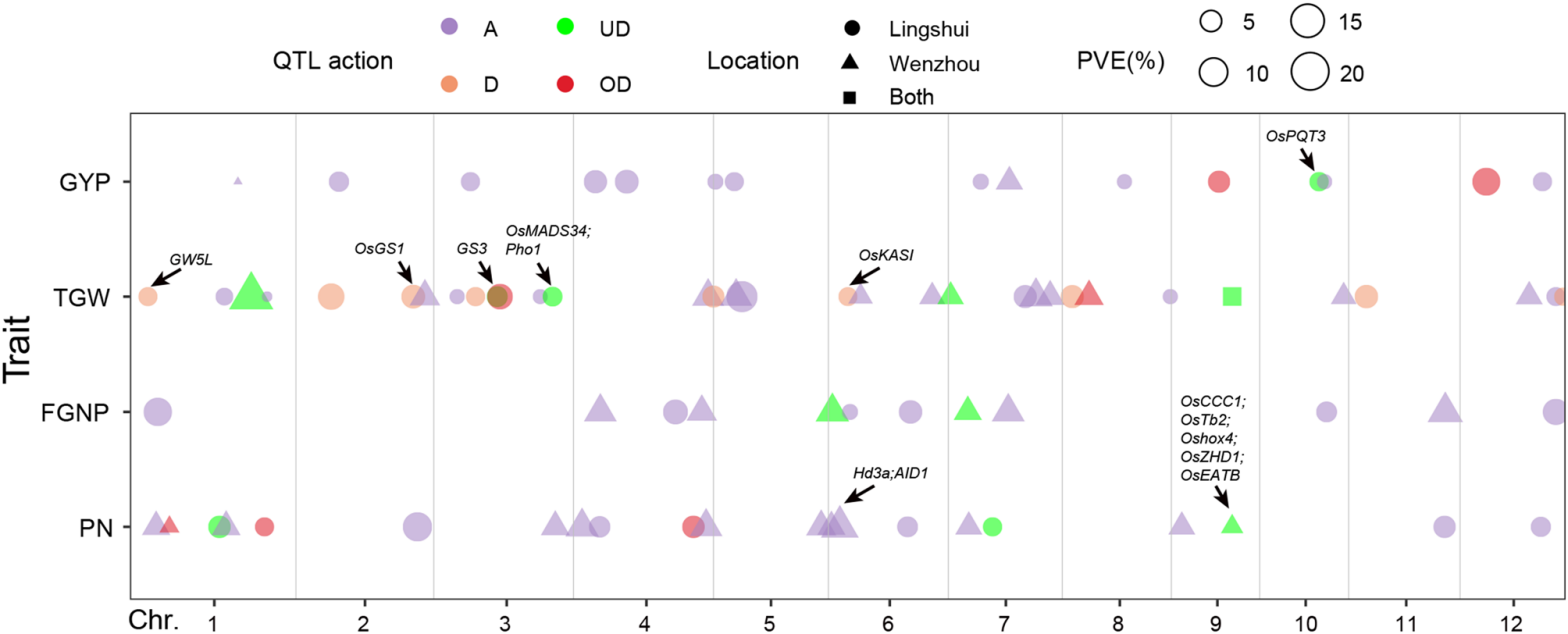
QTLs for four yield-related traits detected in Lines, TCF1s, and for their *H*_*MP*_ in Lingshui and Wenzhou. Symbols above the QTLs represent known genes related to the trait as candidate genes. QTL actions of ‘A’, ‘D’, ‘OD’, and ‘UD’ represent additive, dominance, overdominance, underdominance, respectively. The sizes of the shapes represent the percentage of phenotypic variance explained by the QTL.

A total of 81 main-effect QTLs (M-QTLs) were identified for phenotypic variations of the four traits of the lines (RILs and XSILs), TCF_1_s and absolute mid-parent heterosis (*AH*_MP_) in LS and WZ (Table 3, Fig. 3). The detected M-QTLs were distributed on all the 12 chromosomes, including 54 additive, 10 dominance-type (D-type), 6 overdominance-type (OD-type), and 11 underdominance-type (UD-type) M-QTLs.

**Table 3:** QTLs associated with four yield-related traits in the lines including Xiushui09/IR2061 RILs and Xiushui09 background introgression lines (XSILs), and their testcross F_1_s (PA64S × the RILs and XSILs) in Lingshui (LS) and Wenzhou (WZ).

Trait	QTL	Chr	Interval^1^ (100 kb)	Loc	RILs			RILTCF1			RILTCF1 *AH*_MP_^4^			XSILs			XSILTCF1			XSILTCF1 *AH*_MP_			QTL action	Known genes of yield traits
					LOD	*A*^2^	PVE (%)^3^	LOD	*a* + *d*	PVE (%)	LOD	*d*	PVE (%)	LOD	*a*	PVE (%)	LOD	*a* + *d*	PVE (%)	LOD	*d*	PVE (%)		
PN	*qPN1.1*	1	67.72–69.10	WZ	6.46	1.0	7.0																A	
	*qPN1.2*	1	102.14–104.00	WZ																4.78	4.0	3.1	OD	
	*qPN1.3*	1	233.32–233.67	LS													3.56	− 2.4	8.2	3.30	− 2.1	5.6	UD	
	*qPN1.4*	1	250.54–251.13	WZ										4.37	− 1.2	7.3							A	
	*qPN1.5*	1	350.84–351.01	LS							3.88	1.3	5.5										OD	
	*qPN2*	2	313.56–320.07	LS	8.97	1.2	15.5							4.77	0.7	7.2							A	
	*qPN3*	3	314.46–318.85	WZ										4.61	− 1.1	7.3							A	
	*qPN4.1*	4	21.07–23.45	WZ													6.23	3.6	10.5				A	
	*qPN4.2*	4	66.81–69.68	LS										4.74	1.0	7.3							A	
	*qPN4.3*	4	310.77–314.39	LS				4.54	2.5	5.9	3.29	1.8	5.1	5.45	− 0.8	8.2							OD	
	*qPN4.4*	4	344.45–346.64	WZ										6.18	− 1.1	9.9							A	
	*qPN5*	5	280.21–281.35	WZ	7.58	− 1.1	8.2																A	
	*qPN6.1*	6	7.03–8.70	WZ				3.08	1.4	6.9													A	
	*qPN6.2*	6	17.65–42.03	WZ	9.39	− 1.4	14.2																A	*Hd3a;AID1*
	*qPN6.3*	6	201.12–211.72	LS	4.43	1.1	6.8																A	
	*qPN7.1*	7	52.46–54.43	WZ				3.24	− 1.3	6.3													A	
	*qPN7.2*	7	93.08–137.72	LS							3.39	− 1.3	5.6										UD	
	*qPN9.1*	9	26.93–27.63	WZ													3.55	− 9.6	6.2				A	
	*qPN9.2*	9	137.06–179.79	WZ																3.08	− 8.5	4.0	UD	*OsCCC1;OsTb2; Oshox4; OsZHD1;OsEATB*
	*qPN11*	11	249.00–250.93	LS				6.22	2.0	8.1													A	
	*qPN12*	12	209.42–211.95	LS				4.16	2.1	6.3													A	
FGNP	*qFGNP1*	1	72.14–73.31	LS	7.82	− 14.8	14.5																A	
	*qFGNP4.1*	4	62.71–77.23	WZ													4.76	24.6	9.9				A	
	*qFGNP4.2*	4	259.91–271.56	LS										7.85	13.8	10.5							A	
	*qFGNP4.3*	4	334.09–334.74	WZ	5.56	10.1	8.4																A	
	*qFGNP6.1*	6	8.70–12.07	WZ				4.90	− 18.8	10.6	3.61	− 16.6	7.9										UD	
	*qFGNP6.2*	6	56.02–56.82	LS				7.07	27.5	3.6													A	
	*qFGNP6.3*	6	211.72–216.43	LS	4.52	− 14.8	9.1																A	
	*qFGNP7.1*	7	50.24–51.49	WZ							3.33	− 17.8	7.0										UD	
	*qFGNP7.2*	7	155.40–157.20	WZ										6.23	12.4	10.1							A	
	*qFGNP10*	10	174.33–174.66	LS													3.64	− 25.4	6.8				A	
	*qFGNP11*	11	251.49–252.00	WZ	7.95	− 12.0	12.0																A	
	*qFGNP12*	12	244.56–255.08	LS										6.89	20.0	11.9							A	
TGW	*qTGW1.1*	1	44.86–49.15	LS										5.92	1.0	5.5				3.32	− 0.7	4.8	D	*GW5L*
	*qTGW1.2*	1	245.95–246.72	LS										5.23	− 0.9	4.6							A	
	*qTGW1.3*	1	311.04–321.39	WZ	4.04	− 1.1	4.3	25.83	− 2.6	20.6													UD	
	*qTGW1.4*	1	356.41–358.94	LS													3.06	− 0.6	1.6				A	
	*qTGW2.1*	2	90.07–93.88	LS										12.86	1.0	12.2	4.65	0.6	2.5				D	
	*qTGW2.2*	2	302.91–308.55	LS	7.20	1.0	9.4	10.06	1.3	6.3				3.91	0.6	3.5							D	*OsGS1*
	*qTGW2.3*	2	336.06–336.35	WZ	6.74	1.3	8.4																A	
	*qTGW3.1*	3	60.77–61.07	LS				5.15	0.9	3.2													A	
	*qTGW3.2*	3	102.10–115.58	LS										6.07	0.8	5.6	5.75	0.8	3.1				D	
	*qTGW3.3*	3	164.59–166.28	LS							6.44	− 0.8	6.9										UD	*GS3*
	*qTGW3.4*	3	169.27–175.80	LS							9.05	1.1	11.3										OD	
	*qTGW3.5*	3	273.95–280.84	LS													5.60	− 0.9	3.1				A	
	*qTGW3.6*	3	304.96–314.46	LS																3.99	− 0.7	6.0	UD	*OsMADS34;Pho1*
	*qTGW4.1*	4	350.43–351.30	WZ	8.53	− 1.4	9.5																A	
	*qTGW4.2*	4	364.71–364.73	LS	5.92	− 1.0	7.8	9.78	− 1.3	6.2													D	
	*qTGW5.1*	5	58.67–59.56	WZ										5.46	1.1	9.6							A	
	*qTGW5.2*	5	73.98–74.64	LS													24.88	1.3	18.1				A	
	*qTGW6.1*	6	44.96–56.82	LS	3.01	− 0.7	4.0	6.01	− 1.2	5.4													D	*OsKASI*
	*qTGW6.2*	6	83.54–84.21	WZ				3.15	1.3	5.0													A	
	*qTGW6.3*	6	270.50–270.63	WZ	5.02	1.1	5.6																A	
	*qTGW7.1*	7	0.18–12.20	WZ																3.25	− 1.9	6.0	UD	
	*qTGW7.2*	7	194.56–206.38	LS	6.64	− 1.0	8.8							6.36	− 1.0	5.7							A	
	*qTGW7.3*	7	225.66–231.43	WZ													6.86	3.7	10.6				A	
	*qTGW7.4*	7	246.70–284.29	WZ				4.59	− 1.3	7.4													A	
	*qTGW8.1*	8	22.14–30.32	LS	6.39	1.0	8.8	6.91	− 1.1	4.4													D	
	*qTGW8.2*	8	62.71–77.23	WZ																4.60	3.8	7.7	OD	
	*qTGW8.4*	8	281.23–282.20	LS										3.63	− 0.5	3.2							A	
	*qTGW9*	9	137.06–179.79	LS,WZ							5.35	− 0.8	5.2				3.27	3.0	4.8				UD, A	
	*qTGW10*	10	218.76–219.27	WZ										3.30	1.3	5.5							A	
	*qTGW11*	11	44.40–48.00	LS										5.29	0.6	4.7	15.16	1.0	9.1				D	
	*qTGW12.1*	12	179.75–180.70	WZ										3.74	1.2	6.3							A	
	*qTGW12.2*	12	244.56–255.08	LS	4.28	0.8	5.4																A	
	*qTGW12.3*	12	267.08–271.35	LS										3.01	0.5	2.6	8.75	0.8	5.0				D	
GYP	*qGYP1*	1	232.47–330.71	WZ													4.10	− 16.5	1.2				A	
	*qGYP2*	2	110.59–113.70	LS	4.37	− 2.0	6.4																A	
	*qGYP3*	3	93.12–97.97	LS	3.08	− 1.9	5.6																A	
	*qGYP4.1*	4	56.15–58.14	LS	5.63	2.4	8.9																A	
	*qGYP4.2*	4	118.41–159.17	LS										5.68	3.3	9.5							A	
	*qGYP5.1*	5	3.54–6.58	LS													3.95	− 23.8	3.6				A	
	*qGYP5.2*	5	54.18–54.73	LS										3.57	1.6	5.5							A	
	*qGYP7.1*	7	80.71–88.64	LS				3.03	− 4.6	3.7													A	
	*qGYP7.2*	7	155.40–163.02	WZ				3.25	− 4.0	3.5				4.03	2.9	6.5							A	
	*qGYP8*	8	159.53–164.83	LS													3.04	− 6.1	3.2				A	
	*qGYP9*	9	122.11–126.55	LS				5.16	6.3	7.0	6.46	6.0	8.0										OD	
	*qGYP10.1*	10	153.52–156.13	LS																4.18	− 5.4	5.7	UD	*OsPQT3*
	*qGYP10.2*	10	168.19–170.28	LS													3.22	− 6.6	3.2				A	
	*qGYP12.1*	12	68.21–68.97	LS				3.80	9.1	13.9	3.21	7.4	11.8										OD	
	*qGYP12.2*	12	212.17–217.49	LS										3.61	2.2	5.6							A	

*PN* effective panicle number per plant, *FGNP* filled grain number per panicle, *TGW* 1000-grain weight, *GYP* grain yield per plant, *A* additive, *D* dominance, *OD* overdominance, *U* underdominance.

^1^Interval is based on the Nipponbare reference genome IRGSP 1.0.

^2^In the RILs or XSILs, positive QTL effects were from the Xiushui09 allele. In the testcross F_1_s (TCF_1_s), QTL effects for TCF_1_ performance were estimated by (the heterozygotes—the homozygotes).

^3^Proportion of phenotypic variance explained by the given QTL.

^4^The combined RILs and XSILs were termed as Lines. *AH*_MP_ is the absolute mid-parental heterosis value of the TCF_1_s calculated from *AH*_MP_ = TCF_1_-MP, where MP = (PA64S + Line)/2. In *H*_MP_, the effect refers to the increase of dominance effect when a PA64S/Line heterozygote is replaced with a homozygote. PA64S was replaced by PA64 (an isogenic line of PA64S) for trait measurement because the former shows sterility in later season in WZ.

Nine M-QTLs were identified for PN in LS, explaining 22.3%, 20.3% and 16.4% of the total phenotypic variance in the RILs, RILTCF_1_s and corresponding *AH*_MP_s, and 22.7%, 8.2% and 5.6% of the total phenotypic variance in the XSILs, XSILTCF_1_s and corresponding *AH*_MP_s, respectively. Similarly, 12 M-QTLs were detected for PN in WZ, explaining 29.4% and 13.1% of the total phenotypic variance in the RILs and RILTCF_1_s, and 24.5%, 16.6% and 7.1% of the total phenotypic variance in the XSILs, XSILTCF_1_s and corresponding *AH*_MP_s, respectively. Among them, *qPN1.2* and *qPN1.5* appeared to be OD-type M-QTLs, which increased *AH*_MP_s values by 4.0 in WZ and 1.3 in LS, respectively. The other OD-type M-QTL *qPN4.3* was found in the RILTCF_1_s and corresponding *AH*_MP_s. Three M-QTLs *qPN1.3*, *qPN7.2* and *qPN9.2* appeared to be UD-type QTLs, which decreased *AH*_MP_s values by 2.1 and 1.3 in LS and 8.5 in WZ, respectively. The remaining 15 M-QTLs were detected as additive types, including 8 M-QTLs (*qPN1.1*, *qPN1.4*, *qPN3*, *qPN4.2*, *qPN4.4*, *qPN5*, *qPN6.2* and *qPN6.3*) detected only in the RILs or XSILs, 1 (*qPN2*) both in the RILs and XSILs, and 6 (*qPN4.1*, *qPN6.1*, *qPN7.1*, *qPN9.1*, *qPN11* and *qPN12*) only in the RILTCF_1_s or XSILTCF_1_s. Notably, five genes *Os08g0323700* (*OsCCC1*), *Os09g0410500* (*OsTb2*), *Os09g0470500* (*Oshox4*), *Os09g0466400* (*OsZHD1*) and *Os09g0457900* (*OsEATB*) reportedly contributing to PN were located nearby the UD-type M-QTL *qPN9.2* in WZ, which had a largest negative effect − 8.5 on PN from heterozygote in the TCF_1_s. Moreover, *qPN6.2*, with a negatively additive effect of 1.4 from XS09 allele and the largest logarithm of the odds (LOD) score detected in the lines in WZ, contained two known genes *Os06g0157700* (*Hd3a*) and *Os06g0181300* (*AID1*) for PN.

Of 12 M-QTLs, each six were identified for FGNP in LS and WZ, including ten additive (*qFGNP1*, *qFGNP4.1*, *qFGNP4.2*, *qFGNP4.3*, *qFGNP6.2*, *qFGNP6.3*, *qFGNP7.2*, *qFGNP10*, *qFGNP11* and *qFGNP12*) and two UD-type (*qFGNP6.1* and *qFGNP7.1*) QTLs, which collectively explained 23.6% (20.4%), 3.6% (10.6%) and 0 (14.9%) of the total phenotypic variance in the RILs, RILTCF_1_s and corresponding *AH*_MP_s in LS (WZ), and 22.5% (10.1%) and 6.7% (9.9%) of the total phenotypic variance in the XSILs and XSILTCF_1_s in LS (WZ), respectively. Among them, the UD-type M-QTL *qFGNP7.1* was only detected in the *AH*_MP_s of RILTCF_1_s, with the heterozygote reduced FGNP by 17.8 in WZ. The other UD-type M-QTL *qFGNP6.1*, simultaneously detected in the RILTCF_1_s and corresponding *AH*_MP_s, with a dominance effect of 16.6 for reduced FGNP in WZ.

A total of 33 M-QTLs were detected for TGW, including 20, 12, and 1 QTLs identified only in LS, WZ, and both locations, respectively. These 21 M-QTLs (8 additive, 9 D-type, 1 OD-type and 3 UD-type QTLs) detected in LS together accounting for 44.2% (47.6%), 25.4% (42.6%), and 23.4% (10.8%) of the total phenotypic variance in the RILs (XSILs), RILTCF_1_s (XSILTCF_1_s) and their *AH*_MP_s, respectively. Of them, three (*qTGW1.1*, *qTGW2.2* and *qTGW6.1*) of nine D-type M-QTLs were adjacent to three known TGW-related genes *GW5L*, *OsGS1* and *OsKASI*. Two (*qTGW3.3* and *qTGW3.6*) of three UD-type M-QTLs had a negative overdominance effect causing decreased TGW by 0.82 g and 0.73 g, which co-located or were close to the known TGW-related genes, *GS3*, *OsMADS34* and *Pho1*. In the WZ experiment, 13 TGW M-QTLs (10 additive, 1 OD-type and 2 UD type QTLs) were identified together accounting for 27.9% (21.4%), 33.0% (15.4%) and 13.7% of the total phenotypic variance in the RILs (XSILs), RILTCF_1_s (XSILTCF_1_s) and *AH*_MP_s of XSILTCF_1_s, respectively. Only OD-type M-QTL (*qTGW8.2*) was detectable in *AH*_MP_s of XSILTCF_1_s, with the heterozygote associated with increased TGW by 3.84 g.

Fifteen M-QTLs were detected for GYP, including 13 QTLs (10 additive, 2 OD-type and 1 UD-type) together explained 20.9% (20.6%), 24.6% (10.1%) and 19.7% (5.7%) of the total phenotypic variance in RILs (XSILs), RILTCF_1_s (XSILTCF_1_s) and their *AH*_MP_s in LS, respectively, and 2 additive QTLs explained 6.5% in XSILs and 3.5% (1.2%) in RILTCF_1_s (XSILTCF_1_s) in WZ. Of these, only UD-type QTL (*qGYP10.1*) was identified with a negative dominance effect of 5.4 g in LS, which contained a known gene *OsPQT3* controlling rice grain yield in the field conditions. In addition, two OD-type M-QTLs *qGYP9* and *qGYP12.1* were only detected in *AH*_MP_s of RILTCF_1_s and both with positive dominance effects of 6.0 g and 7.4 g in WZ.

Not any co-located QTL was detected among 32 QTLs for the traits in the RIL and the *AH*_MP_ populations at the two locations, and only one co-located QTL (*qTGW1.1*) was detected at LS among 34 QTLs for the traits in the XSIL and the *AH*_MP_ populations (Table 3).

### Epistatic QTL mapping of yield-related traits and mid-parent heterosis

In order to test whether these M-QTLs had epistatic effects on the relevant traits, we checked all epistatic QTL (E-QTL) pairs identified in the RILs or XSILs, their TCF_1_s and *AH*_MP_s. Eight, 21 and 12 E-QTL pairs were identified in line, TCF_1_ and *AH*_*MP*_ populations, respectively, which involved one M-QTL and the other random loci (Table 4). For PN, 3 and 4 E-QTLs in the RILs and XSILs, 6 E-QTLs in XSILTCF_1_s, and 4 E-QTLs in *AH*_MP_s of XSILTCF_1_s were identified. The total phenotypic variation explained (PVE) of E-QTLs for PN was 6.84% for RILs in LS and 11.10% for XSILs in WZ, 6.32% for XSILTCF_1_s in WZ, and 6.40% and 1.12% for *AH*_MP_s of XSILTCF_1_s in LS and WZ, respectively. For GYP, only one E-QTL in the RILs, 15 E-QTLs in XSILTCF_1_s, and 8 E-QTLs in *AH*_MP_s of XSILTCF_1_s were identified. The total PVE of E-QTLs for GYP was 13.05% for RILs in LS, 3.52% for XSILTCF_1_s in WZ, and 1.86% for *AH*_MP_s in WZ. Among M-QTLs involved epistasis on GYP and PN, *qGYP1* and *qPN1.3* on chromosome 1 were the most important loci contributing to the TCF_1_ performances and heterosis of GYP and PN both in XSIL and the corresponding *AH*_*MP*_ populations. No E-QTLs were identified for TGW and FGNP. No E-QTL between two M-QTLs was observed.

**Table 4:** Digenic epistatic QTLs affecting four yield-related traits detected in the Xiushui09/IR2061 RILs and Xiushui09 background introgression lines (XSILs), and their testcross F1s (PA64S × the RILs and XSILs) in Lingshui (LS) and Wenzhou (WZ).

Pop	Trait^1^	Loc	Chr	Interval^2^ *i* (100 kb)	Chr	Interval *j* (100 kb)	Lines					TCF1s					*AH*_MP_				
							LOD	*A*_*i*_^2^	*A*_*j*_^2^	*AA*_*ij*_^3^	PVE(%)^4^	LOD	*A*_*i*_	*A*_*j*_	*AA*_*ij*_	PVE(%)	LOD	*A*_*i*_	*A*_*j*_	*AA*_*ij*_	PVE(%)
RIL	GYP	LS	2	103.47–104.38	2	**110.59–113.70**	5.93	0.2	− 1.2	− 4.7	13.05										
XSIL	GYP	WZ	1	21.07–23.45	1	**326.81–327.74**						6.70	− 28.3	− 22.1	− 19.9	0.27					
XSIL	GYP	WZ	1	**326.81–327.74**	2	336.35–338.16						8.98	0.7	26.0	28.0	0.22					
XSIL	GYP	WZ	1	**326.81–327.74**	3	300.2–303.79						9.26	− 2.5	27.7	20.6	0.27					
XSIL	GYP	WZ	1	**326.81–327.74**	4	210.07–213.11						10.4	0.9	27.4	31.8	0.23					
XSIL	GYP	WZ	1	**326.81–327.74**	5	258.18–258.42						9.15	− 0.2	27.3	28.6	0.21					
XSIL	GYP	WZ	1	**326.81–327.74**	6	16.33–17.56						11.15	1.5	27.2	32.6	0.24					
XSIL	GYP	WZ	1	**326.81–327.74**	7	246.7–284.29						9.54	− 30.6	− 27.6	− 31.3	0.22					
XSIL	GYP	WZ	1	**326.81–327.74**	8	5.59–6.56						5.94	− 23.2	− 24.0	− 24.1	0.22					
XSIL	GYP	WZ	1	**326.81–327.74**	9	109.44–113.72						8.40	− 24.3	− 25.5	− 23.0	0.22					
XSIL	GYP	WZ	1	**326.81–327.74**	10	200.12–203.29						9.21	− 27.5	− 25.0	− 30.2	0.26					
XSIL	GYP	WZ	1	**326.81–327.74**	11	70.73–71.45						7.12	− 23.9	− 24.5	− 24.2	0.22					
XSIL	GYP	WZ	1	**326.81–327.74**	12	244.56–255.08						9.50	− 25.9	− 27.3	− 24.8	0.22					
XSIL	GYP	WZ	1	21.07–23.45	1	**326.81–327.74**											7.74	− 30.4	− 32.8	− 32.9	0.22
XSIL	GYP	WZ	1	**326.81–327.74**	5	258.18–258.42											7.95	− 0.5	30.8	30.6	0.21
XSIL	GYP	WZ	1	**326.81–327.74**	6	16.33–17.56											11.02	1.4	30.1	37.5	0.27
XSIL	GYP	WZ	1	**326.81–327.74**	12	151.55–154.08											9.35	− 0.3	30.2	32.3	0.22
XSIL	GYP	WZ	4	306.09–309.39	7	**155.4–157.2**						9.99	− 7.7	− 4.3	− 35.5	0.28					
XSIL	GYP	WZ	4	306.09–309.39	7	**155.40–157.20**											7.84	− 7.6	− 5.1	− 37.4	0.3
XSIL	GYP	WZ	4	258.23–259.91	8	**91.36–159.53**											6.36	2.5	37.0	38.5	0.12
XSIL	GYP	WZ	6	16.33–17.56	12	**244.56–255.08**						11.90	25.4	− 0.9	29.7	0.23					
XSIL	GYP	WZ	6	16.33–17.56	8	**91.36–159.53**											10.78	− 7	− 1.5	− 36.8	0.27
XSIL	GYP	WZ	7	**155.40–157.20**	10	200.12–203.29						5.49	− 4.0	− 0.6	− 23.7	0.21					
XSIL	GYP	WZ	7	**155.40–157.20**	9	209.64–213.92											7.64	− 2.3	4.9	− 27.1	0.25
RIL	PN	LS	3	161.99–162.53	5	**280.21–281.35**	5.95	0.6	− 0.8	− 1.0	1.53										
RIL	PN	LS	6	12.07–16.33	6	**201.12–201.59**	9.44	− 1.7	2.2	− 2.0	3.19										
RIL	PN	LS	6	114.77–114.82	12	**209.42–211.95**	8.21	2.8	− 3.1	− 3.2	2.12										
XSIL	PN	WZ	1	26.93–27.63	7	**155.4–157.2**						8.43	0	− 10.8	− 11.5	1.15					
XSIL	PN	WZ	1	**233.32–233.67**	10	200.12–203.29						5.03	1.0	− 0.7	10.3	0.72					
XSIL	PN	WZ	1	**233.32–233.67**	11	26.7–28.83						6.39	1.2	0.9	11.9	0.73					
XSIL	PN	LS	1	**233.32–233.67**	5	140.65–148.26											5.66	− 7.8	− 0.1	− 7.0	2.82
XSIL	PN	LS	1	**233.32–233.67**	6	221.57–223.16											5.48	− 0.6	1.0	7.9	3.58
XSIL	PN	WZ	1	**233.32–233.67**	5	140.65–148.26											5.19	− 8.4	− 0.5	− 9.7	0.56
XSIL	PN	WZ	3	**314.46–318.85**	7	155.4–157.2	5.52	− 2.3	− 1.3	1.8	4.06										
XSIL	PN	WZ	4	306.09–309.39	12	**209.42–211.95**	5.75	− 1.9	− 2.2	2.2	1.92										
XSIL	PN	WZ	6	**204.13–205.05**	6	221.57–223.16						6.08	− 0.3	− 9.5	− 10.4	1.22					
XSIL	PN	WZ	6	**204.13–205.05**	7	155.4–157.2						7.15	− 0.5	− 8.2	− 9.2	1.30					
XSIL	PN	WZ	6	**204.13–205.05**	12	151.55–154.08						6.78	− 0.7	− 10.4	− 10.7	1.20					
XSIL	PN	WZ	6	**204.13–205.05**	6	308.28–308.62	5.62	− 1.5	− 1.9	2.3	3.39										
XSIL	PN	WZ	6	308.28–308.62	9	**8.3–59.36**	5.05	− 2.0	− 2.1	2.1	1.73										
XSIL	PN	WZ	9	**137.06–179.79**	12	267.08–271.35											5.50	1.4	0.2	10.1	0.56

^1^*PN* effective panicle number per plant, *GYP* grain yield per plant.

^2^Interval is based on the Nipponbare reference genome IRGSP 1.0

^3^*A*_i_ and *A*_j_ are the main effects of locus *i* and locus *j*. *AA*_*ij*_ is the epistatic effect between loci *i* and *j*, as defined by Mei et al.^69^. For RILs or XSILs, the main effects of the loci *i* and *j*, arising from the substitution of the IR2061 allele by the XS09 allele. For TCF_1_s and *H*_MP_, the main effects of the loci *i* and *j*, estimated by the difference between heterozygote (PA64S/XS09) and homozygote using the mean F_1_ and *H*_MP_ values.

^4^Percentage of the total variation explained by *AA*_*ij*_*.*

Bold markers are those flanking M-QTLs identified in Table 3.

## Discussion

QTLs play an important role to enrich our understanding of the genetic basis of heterosis in rice. This study permitted the direct measurement of heterosis for all the measured traits using two sets of lines (RILs and XSILs) together with their TCF_1_s and H_MP_s that exaggerated the capacity to more precisely resolve different types of gene actions for identified QTLs that were responsible for trait performance and heterosis.

In this study, the phenotypic performance of four yield traits was evaluated in two locations, LS and WZ. The performance of hybrid plants was mainly determined by their mid-parental value and the heterosis level. In both the locations, the XSILTCF_1_s had higher *H*_MPS_ of PN, FGNP, and GYP but lower TGW than the RILTCF_1_s, which suggested that the portion of introgression of IR2061 (*indica*)’s genomic fragment into XS09 (*japonica*) background may influence the effect of heterosis. Recently, Lin et al. reported that the introduction of *japonica* germplasm played an important role in *indica* hybrid breeding^36^. In their study, only 3.31% of the genome in the parents of the *indica* hybrids were contributed by *japonica* germplasm, which affected about half of the grain yield heterotic loci^36^. To evaluate the effects of the proportion of *indica* and *japonica* genome on heterosis, we explored the correlations between the ratios of heterozygous or homozygous XS09 alleles and the heterosis for four yield-related traits (Fig. 2). We observed a weak trend for higher ratios of homozygous XS09 genotype in RILs/XSILs or heterozygous genotype in their TCF_1_s with higher heterosis for most yield-related traits except for TGW. This trend is even more pronounced in the combined line populations (RILs and XSILs). Indeed, moderate genomic differences between parents of *indica*–*japonica* cross do improve, at least to some extent, grain yield and its degree of heterosis in rice^30,37,38^. However, the genomic differences between parents of *indica*–*japonica* cross for attaining strongest heterosis varies according to the cross, and actually there is no a fixed proportion of introgression of *indica* genome into *japonica* background or *japonica* genome into *indica* background for the parents, as indicated by *indica*–*japonica* hybrid breeding practices in China^37,39,40^.

Our study provides a possibility to identify significant genetic factors for heterosis by comparing QTLs detected from different datasets. A total of 81 M-QTLs were identified for phenotypic variation of four traits of lines (RILs and XSILs) or TCF_1_s and *AH*_MP_ values in both locations. The most striking finding was the presence of two predominant types of M-QTLs for yield-related traits except for TGW, the additive M-QTLs and OD/UD-type M-QTLs, without M-QTLs exhibiting complete and partial dominance (Fig. 3, Table 3). Previously, it has been reported that the genetic basis of heterosis is mainly determined by dominance and overdominance effects^14,17,41^. Furthermore, Wen et al. used F_1_ hybrids in the NCII design to dissect the genetic basis of heterosis by investigating the factors that mainly affect the heterosis were dominance, dominance-by-dominance, overdominance, and complete dominance QTL^42^. Here, 83.3%, 80.0%, 71.4% and 16.7%, 20.0%, 23.8% of the detected M-QTLs were attributed by additive QTL actions and OD/UD-type QTL actions in FGNP, GYP and PN, respectively. However, 9 of 10 D-type M-QTLs in this study were detected in TGW, which may result in a relatively low proportion (51.5%) of the detected 33 M-QTLs for TGW with additive QTL actions. On the other hand, it is worth noting that 13 of 81 M-QTLs were also detected as E-QTLs with another random locus without significant M-QTL. Our results confirmed the previous reports of epistasis and overdominance as the major genetic basis of heterosis in rice^8,43^, and these findings are also consistent with Li et al.^44^ and Melchinger et al.^45^. However, almost no genetic overlap was found between the QTLs affecting the traits in inbred line populations (RILs and XS09) and the ones underlying heterosis in their testcross populations (XSILTCF_1_s and RILTCF_1_s) under the two locations, suggesting that different genetic mechanisms involved in trait itself and its heterosis.

In the present study, GYP showed a strongest heterosis among the four yield-related traits studied, and TGW had a weakest heterosis in XSILTCF_1_s and RILTCF_1_s (Table 1). Heterosis for GYP in TCF_1_s was mainly attributed to yield-component traits FGNP and PN both in the two sets of testcrosses, consistent with the findings of previous studies performed on rice^8,30,37,43^. In TCF_1_s, the heterosis of GYP and yield component traits (PN and FGNP) was mainly produced by the overdominance of heterotic loci, indicating that non-additive gene actions are pivotal to grain yield. This finding is consistent with He et al. as they used RILs based NCII design for E-QTLs to estimate genomic position, digenic interactions of QTL, additive and dominance effects, and they noted that non-additive gene actions mainly contribute to heterosis^46^. Further, we explored the correlations among the lines, TCF_1_s and *H*_MP_s, and found highly positive correlations between TCF_1_s and *H*_MP_s and lower positive correlations between the lines and TCF_1_s for FGNP and GYP in both locations (Table 2). These results suggested that non-additive QTL was a contributor to TCF_1_s for FGNP and GYP traits. Contrarily, a high positive correlation was found between the lines and TCF_1_s for TGW compared to the low positive correlation between TCF_1_s and *H*_MP_s in LS, indicating that additive QTL mainly contribute to TCF_1_s for TGW only in LS. However, the correlations between the lines and TCF_1_s for TGW in WZ exhibited contradictory results as indicted in LS. The negative correlation between the lines and their *H*_MP_s evidently indicated that additive and dominant QTLs acted independently in the testcross populations as previously reported^17^. In our study, the correlations between the lines and *H*_MP_s for yield traits were different according to genetic background and location, which suggested that the role of heterotic loci for yield was affected by genetic background and environment^8,41,47,48^. Actually, a lot of epistasis between two random loci were detected for the four traits under the two locations (data not shown). So, complexity of heterosis in rice, reflected by a large number of loci involved, complex epistatic relationships, and genetic background- and environment-dependent gene actions on heterosis, suggested that marker-assisted selection for significantly improving heterosis of yield traits in hybrid rice breeding programs may be very challenging.

A few already reported genes in rice were co-located with the heterotic QTLs identified in this study such as *qGYP10.1* (*OsPQT3*, increases grain yield in the field)^49^, *qPN9.2* and *qTGW9* (*OsCCC1* for cell elongation and panicle number^50^, *OsTb2* for tillering and grain yield per panicle^51^, and *Oshox4* affecting bushy tillers^52^, *OsZHD1* for tiller number^53^, and *OsEATB*, ERF protein associated with tillering and panicle branching^54^, *qTGW1.1* (*GW5L*, negatively regulates grain width and weight)^55^, *qTGW2.2* (*OsGS1*, growth rate and grain filling)^56^, *qTGW3.3* (*GS3*, grain length and weight)^57^, *qTGW3.6* (*OsMADS34*, grain size and yield^58^, *Pho1*, the size of mature seeds and the starch content)^59^, and *qTGW6.1* (*OsKASI*, 1000-grain weight and tiller number)^60^. All the above mentioned genes provided heterotic effects with heterozygous alleles in UD-type or D-type M-QTLs. However, some M-QTLs detected in this study exhibited a positive overdominance heterosis such as *qPN1.2*, *qPN1.5* and *qPN4.3* for increased *AH*_MP_s of PN in TCF_1_s; *qGYP9* and *qGYP12.1* for positive overdominance effect of GYP in both TCF_1_s and *AH*_MP_s. They enhanced the performance of TCF_1_s hybrid by increasing PN and GYP. Similarly, *qTGW3.4* and *qTGW8.2* enhanced TGW and showed a positive overdominance effect in *AH*_MP_s. These overdominance heterotic QTLs identified in this study across different populations would be highly valuable for breeding to enhance grain yield of hybrid rice by marker-assisted selection.

## Materials and methods

### Experimental materials

The rice populations used in this study included a set of 209 F_2_:_10_ RILs derived from single-seed descent from a cross between a photosensitive late *japonica* variety XS09 developed in China and an *indica* inbred line IR2061 developed at IRRI, and a set of 222 BC_2_F_8_ XSILs under XS09 background with IR2061 as a donor. Then two testcross F_1_ populations were developed by crossing the RILs and XSILs to a common maternal tester line PA64S, which is a stable *indica* thermo-sensitive genic male sterile line with excellent wide compatibility to both *indica* and *japonica* cultivars^61^ and has been extensively used as a sterile line in two-line hybrid rice breeding programs in China. The first testcross population consisting of 209 RILTCF_1_s from crosses between the RILs (used as male) and PA64S. The second one consisted of 222 XSILTCF_1_s from crosses between the XSILs (used as male) and PA64S. In addition, the parents XS09 and IR2061, PA64 (the recurrent parent of PA64S, an isogenic line of PA64S), two F_1_s (XS09 × IR2061 and PA64S × XS09) were used as checks in the phenotyping experiments.

### Field experiment and trait evaluation

Field experiments were conducted at two experimental stations of Wenzhou Academy of Agricultural Sciences, including LS (18.5° N, 110° E) of Hainan Province and WZ (27.3° N, 119.4° E) of Zhejiang Province. In the LS experiment, the RILs, XSILs, the two testcross populations (RILTCF_1_s and XSILTCF_1_s), the parents (XS09, IR2061 and PA64S) and their F_1_s (XS09 × IR2061 and PA64S × XS09) were sowed in the seedling nursery on November 25, 2014. The 25-day-old seedlings were transplanted into four-row plots each consisting of a single row of the male RIL, XSIL and the two testcross hybrids (RILTCF_1_ and XSILTCF_1_). The plots were arranged in a randomized complete block design with three replications. Each row within a plot consisted of 12 plants with a spacing of 17 cm between the plants and 25 cm between rows. Five check plots consisting of XS09, IR2061, PA64S, and XS09 × IR2061 F_1_ and PA64S × XS09 F_1_ were randomly arranged in each replication. In the WZ experiment, materials were sowed in the seedling nursery on June 15, 2015, and the 25-day-old seedlings were transplanted into four-row plots each consisting of a single row of a RIL, XSIL and the two testcross hybrids. The field arrangement in WZ was the same as the LS experiment. In addition, five check plots consisting of XS09, IR2061, PA64, XS09 × IR2061 F_1_, and PA64S × XS09 F_1_ were included in each replication. Crop management followed local field production practices in the two sites. At maturity, PN was investigated from the middle 10 plants. Total grain number per panicle was measured from 10 main panicles from the middle 10 plants (one main panicle each plant) each plot. The grain yield each plot was obtained after weighing all grains collected from the rest panicles and the 10 main panicles of the middle 10 plants. Then GYP (g) was calculated by the ratio of grain yield each plot to 10. FGNP was calculated by the ratio filled grain number each plot to 10. An estimate of the TGW (g) was made by weighing three lots of 100 grains per entry.

For each testcross F_1_, *AH*_MP_ and the relative *H*_MP_ was calculated as *AH*_MP_ = F_1_ − MP and *H*_MP_ (%) = (F_1_ − MP)/MP × 100, respectively, where F_1_ is the trait value of a testcross F_1_ and MP is the mean value of the corresponding paternal RIL or XSIL and the common maternal tester line PA64S in LS and PA64 (an isogenic line of PA64S) in WZ. PA64S was replaced by PA64 for trait measurement because the former shows sterility in later season in WZ, where the temperature is over 23.5 °C at the panicle differentiation stage (a crucial stage of fertility transformation of two-line sterile lines.

### SNP genotyping

Genomic DNA for SNP genotyping was isolated from approximately 100 mg fresh leaf samples of 5-week-old seedlings for the 209 RILs, 222 XSILs, the parents XS09 and IR2061, and a tester line PA64S using a modified cetyltrimethylammonium bromide (CTAB) method^62^. Genotyping was performed using a customized rice 56 K SNP array containing 56,897 SNP screened from the 3 K Rice Genome Project^63,64^. Target DNA preparation, chip hybridization, and array processing were conducted by CapitalBio Technology (Beijing, China) according to the Affymetrix Axiom 2.0 assay protocol. A total of 39,070 high-quality SNPs was screened based on polymorphism between XS09 and IR2061. Among them, 25,296 high-quality non-redundant SNPs were finally selected for genotype analysis (Fig. S1).

Genotypes of TCF_1_s at 25,296 SNPs were determined based on the SNP genotypes of the corresponding RILs or XSILs and PA64S. Specifically, if the two parents (RIL or XSIL and PA64S) have the same homozygous genotype, their TCF_1_s shared the same genotype, and if the two parents have different homozygous genotypes, the genotypes of their TCF_1_s were deduced as heterozygotes.

### Genetic linkage map construction and QTL mapping

Filtered and high-quality SNPs with less than 10% missing were used for the construction of bin maps for each population using the BIN function in QTL IciMapping Version 4.2^65^. For non-redundancy, only one SNP was retained to represent each bin, either one with a minimum missing rate, or a random one when the missing rate was equal. The SNPs which displayed a unique pattern of segregation and did not fall into a bin were removed. We then constructed the linkage map of each population using these bins by the MAP function in QTL IciMapping Version 4.2^65^. The values obtained for the recombination frequencies were converted into map distance by the Kosambi mapping function^66^. A total of 1756 bins were used to construct two high-density linkage maps with 2017.1 cM and 1082.9 cM for RIL and XSIL populations, respectively. The genotypes for each cross in the RILTCF_1_s and XSILTCF_1_s were deduced from the RILs/XSILs and the original parents that were used as the parents for the crosses.

QTL mapping for four yield-related traits was performed separately for the RIL, XSIL, RILTCF_1_, and XSILTCF_1_ populations. For the RIL and XSIL datasets, the mean trait values from three replications at each location were used as input data. For RILTCF_1_ and XSILTCF_1_ populations, the mean trait values and *AH*_MP_s of the TCF_1_s were used as input data. All datasets were analyzed using the biparental populations (BIP) function in QTL IciMapping Version 4.2^65^. The inclusive composite interval mapping of additive (ICIM-ADD) QTL method was performed to identify M-QTLs by using default settings. The analyses of M-QTLs were performed with pre-adjusted IciMapping parameters, in which the *P* values for entering a variable (PIN) were set at 0.001 and the scanning step was set at 1.0 cM. The inclusive composite interval mapping of the digenic epistatic (ICIM-EPI) QTL method was used to find possible digenic E-QTLs by using default settings. The corresponding scan step and PIN for E-QTLs mapping were set at 5 cM and 0.0001, respectively. The LOD threshold values 3.0 and 5.0 were used to declare significant M-QTLs and E-QTLs, respectively. The physical position of a QTL was retrieved based on the left and right markers of the detected interval. The known genes underlying the related traits within an identified QTL interval were considered as candidate genes based on the Nipponbare reference genome (IRGSP 1.0)^67^. The QTLs were named as “q + trait abbreviation + chromosome number + QTL number” following the rules recommended by McCouch and CGSN^68^. The type of digenic epistasis without M-QTL was ignored.

### Inference of M-QTL actions

The detected M-QTLs can be divided into four types by estimating additive effect and dominance effect in RILs or XSILs and corresponding TCF_1_s according to the Mei et al.’s and Kim et al.’s methods^18,69^: additive (detected only in lines or TCF_1_s) QTLs, heterotic (D-type, OD-type, and UD-type) M-QTLs. D-type and OD-type QTLs were determined using the values of d/a and a + d. QTLs with 0 <|d/a|< 1 or |2d/(a + d)|≤ 1 or |2a/(a + d)|≥ 1 were designated as D-type QTLs. QTLs with 0 <|d/a|< 1 or |2d/(a + d)|> 1 or |2a/(a + d)|< 1, or those detected only in *AH*_MP_ datasets, were designated as OD-type QTLs. QTLs detected in *AH*_MP_ datasets but showing negative ‘d’ values were defined as UD-type QTLs. Of the detected QTLs, only D-type, OD-type, and UD-type M-QTLs were used for subsequent comparative studies and direct effect analysis. In this study, all these heterotic QTLs are genetic loci underlying heterosis of yield-related traits in rice.

### Statistical analysis

Analyses of variance were performed to determine significant variation between locations and genotypes for all measured traits by the Agricolae Package in R. Significant phenotypic differences among the check parents and the relative hybrids using Duncan’s multiple comparison test, and among RILs, XSILs and the TCF_1_s were statistically assessed using Student’s *t*-test by the agricolae package in R. Here, RILs and XSILs when combined were termed “lines”. *H*_MP_ was tested with a Student’s *t*-test based on the contrast between F_1_ hybrid mean and average performance of corresponding parental lines^70^. Pearson’s correlation analyses among the phenotypic traits measured were performed by the Hmisc Package in R.

### Legislation statement

The experimental research and field studies on plant materials comply with relevant institutional, national, and international guidelines and legislation.

## Supplementary Information

Supplementary Information.

## Abbreviations

LS: Lingshui
WZ: Wenzhou
XS09: Xiushui09
PA64S: Peiai64S
PN: Panicle number per plant
FGNP: Filled grain number per panicle
TGW: 1000-Grain weight
GYP: Grain yield per plant
RIL: Recombinant inbred line
XSIL: Xiushui09 background introgression line
TCF_1_: Testcross F_1_
RILTCF_1_s: Testcross F_1_s of RILs with PA64S
XSILTCF_1_s: Testcross F_1_s of XSILs with PA64S
*H*_*MP*_: Mid-parent heterosis
*AH*_MP_: Absolute mid-parent heterosis
QTL: Quantitative trait locus
M-QTL: Main-effect QTL
E-QTL: Epistatic QTL
D-type: Dominance-type
OD-type: Overdominance-type
UD-type: Underdominance-type
PVE: Phenotypic variation explained
SNP: Single nucleotide polymorphism
LOD: Logarithm of the odds
