# Supplementary material for: Genetic dissection of heterosis of indica–japonica by introgression line, recombinant inbred line and their testcross populations
Source: Sci Rep. 2021 May 13;11:10265. doi: 10.1038/s41598-021-89691-6 (PMC8119717; doi:10.1038/s41598-021-89691-6)
Supplement: Supplementary file 1 — Supplementary Information. [file 41598_2021_89691_MOESM1_ESM.docx]

Genetic dissection of heterosis of *indica*-*japonica* by introgression line, recombinant inbred line and their testcross populations

Wenqing Yang^1†^, Fan Zhang^2†^, Sundus Zafar^3†^, Junmin Wang^4^, Huajin Lu^1^, Shahzad Naveed^2^, Jue Lou^1*^, Jianlong Xu^2*^

^1^ Southern Zhejiang Key Laboratory of Crop Breeding, Wenzhou Vocational College of Science and Technology, Wenzhou, Zhejiang 325006, China；

[yangwq157157@163.com](mailto:yangwq157157@163.com) (W.Y.); [luhjzw@163.com](mailto:luhjzw@163.com) (H.L.); [loujue409@163.com](mailto:loujue409@163.com) J.L.)

^2^ Institute of Crop Sciences/National Key Facility for Crop Gene Resources and Genetic Improvement, Chinese Academy of Agricultural Sciences, Beijing 100081, China

[zhangfan03@caas.cn](mailto:zhangfan03@caas.cn) (F.Z.); [shahzad.nibge@gmail.com](mailto:shahzad.nibge@gmail.com) (S.N.); [xujlcaas@126.com](mailto:xujlcaas@126.com) (J.X.)

^3^ Shenzhen Branch, Guangdong Laboratory for Lingnan Modern Agriculture, Agricultural Genomics Institute at Shenzhen, Chinese Academy of Agricultural Sciences, Shenzhen 518120, China

[sundus_zafar@yahoo.com](mailto:sundus_zafar@yahoo.com) (S.Z.)

^4^ The Institute of Crops and Nuclear Technology Utilization, Zhejiang Academy of Agricultural Sciences, Hangzhou 310021, Zhejiang, China

[wangjm917@sina.com](mailto:wangjm917@sina.com) (J.W.)

* Correspondence: loujue409@163.com (J.L.); xujlcaas@126.com (J.X.)

† These authors contributed equally to this work.

**Supplementary information**

| **Location** | **Population ^1^** | **Item ^2^** | **PN ^3^** | **FGNP** | **TGW** | **GYP** |
| --- | --- | --- | --- | --- | --- | --- |
| Lingshui | RILs | TCF1 trait value vs homozygous genotype ratio in RILs/XSILs | 0.341** | -0.12 | -0.091 | -0.162* |
|  |  | TCF1 trait value vs heterozygous genotype ratio in TCF1s | 0.225** | -0.162* | -0.039 | -0.156* |
|  |  | TCF1 trait value vs homozygous genotype ratio in TCF1s | 0.341** | -0.082 | -0.104 | -0.14* |
|  |  | *H_MP_* vs homozygous genotype ratio in RILs/XSILs | 0.214** | 0.149* | -0.203** | -0.089 |
|  |  | *H_MP_* vs heterozygous genotype ratio in TCF1s | 0.138* | 0.133 | -0.241** | -0.039 |
|  |  | *H_MP_* vs homozygous genotype ratio in TCF1s | 0.217** | 0.132 | -0.149* | -0.099 |
|  | XSILs | TCF1 trait value vs homozygous genotype ratio in RILs/XSILs | -0.05 | 0.075 | 0.308** | 0.077 |
|  |  | TCF1 trait value vs heterozygous genotype ratio in TCF1s | -0.02 | -0.111 | 0.281** | -0.1 |
|  |  | TCF1 trait value vs homozygous genotype ratio in TCF1s | -0.041 | 0.157* | 0.144* | 0.153* |
|  |  | *H_MP_* vs homozygous genotype ratio in RILs/XSILs | 0.014 | -0.04 | -0.024 | -0.03 |
|  |  | *H_MP_* vs heterozygous genotype ratio in TCF1s | 0.049 | 0.069 | -0.087 | -0.006 |
|  |  | *H_MP_* vs homozygous genotype ratio in TCF1s | -0.02 | -0.093 | 0.024 | -0.032 |
|  | RILs and XSILs | TCF1 trait value vs homozygous genotype ratio in RILs/XSILs | 0.386** | 0.065 | -0.505** | -0.077 |
|  |  | TCF1 trait value vs heterozygous genotype ratio in TCF1s | 0.343** | 0.017 | -0.451** | -0.101* |
|  |  | TCF1 trait value vs homozygous genotype ratio in TCF1s | 0.387** | 0.084 | -0.507** | -0.063 |
|  |  | *H_MP_* vs homozygous genotype ratio in RILs/XSILs | 0.321** | 0.318** | -0.556** | 0.1* |
|  |  | *H_MP_* vs heterozygous genotype ratio in TCF1s | 0.294** | 0.316** | -0.556** | 0.108* |
|  |  | *H_MP_* vs homozygous genotype ratio in TCF1s | 0.319** | 0.303** | -0.53** | 0.092 |
| Wenzhou | RILs | TCF1 trait value vs homozygous genotype ratio in RILs/XSILs | 0.122 | -0.007 | -0.062 | -0.047 |
|  |  | TCF1 trait value vs heterozygous genotype ratio in TCF1s | 0.096 | -0.033 | -0.074 | -0.053 |
|  |  | TCF1 trait value vs homozygous genotype ratio in TCF1s | 0.113 | 0.008 | -0.046 | -0.039 |
|  |  | *H_MP_* vs homozygous genotype ratio in RILs/XSILs | 0.045 | 0.124 | 0.08 | 0.073 |
|  |  | *H_MP_* vs heterozygous genotype ratio in TCF1s | 0.015 | 0.114 | 0.033 | 0.11 |
|  |  | *H_MP_* vs homozygous genotype ratio in TCF1s | 0.052 | 0.112 | 0.09 | 0.043 |
|  | XSILs | TCF1 trait value vs homozygous genotype ratio in RILs/XSILs | -0.04 | 0.162* | -0.029 | 0.048 |
|  |  | TCF1 trait value vs heterozygous genotype ratio in TCF1s | -0.004 | 0.083 | 0.097 | 0.104 |
|  |  | TCF1 trait value vs homozygous genotype ratio in TCF1s | -0.048 | 0.116 | -0.101 | -0.011 |
|  |  | *H_MP_* vs homozygous genotype ratio in RILs/XSILs | -0.011 | 0.039 | -0.044 | 0.002 |
|  |  | *H_MP_* vs heterozygous genotype ratio in TCF1s | 0.052 | 0.174** | 0.072 | 0.185** |
|  |  | *H_MP_* vs homozygous genotype ratio in TCF1s | -0.052 | -0.075 | -0.095 | -0.112 |
|  | RILs and XSILs | TCF1 trait value vs homozygous genotype ratio in RILs/XSILs | 0.200** | 0.325** | -0.546** | 0.075 |
|  |  | TCF1 trait value vs heterozygous genotype ratio in TCF1s | 0.190** | 0.298** | -0.504** | 0.081 |
|  |  | TCF1 trait value vs homozygous genotype ratio in TCF1s | 0.194** | 0.322** | -0.54** | 0.069 |
|  |  | *H_MP_* vs homozygous genotype ratio in RILs/XSILs | 0.318** | 0.235** | -0.158** | 0.198** |
|  |  | *H_MP_* vs heterozygous genotype ratio in TCF1s | 0.303** | 0.249** | -0.141** | 0.235** |
|  |  | *H_MP_* vs homozygous genotype ratio in TCF1s | 0.309** | 0.218** | -0.158** | 0.173** |

**Table S1.** Correlation coefficients between the ratio of the heterozygous and homozygous genotype of rice individuals and the value of relative TCF1s and mid-parent heterosis (*H_MP_*) in Lingshui and Wenzhou

^1^ RILs: recombinant inbred lines derived from a cross between Xiushui09 and IR2061; XSILs: introgression lines under Xiushui09 background with IR2061 as a donor;

^2^ *H_MP_*, mid-parent heterosis.

^3^ PN, effective panicle number per plant; FGNP, filled grain number per panicle; TGW, 1000-grain weight; GYP, grain yield per plant. ‘∗’ and ‘∗∗’ indicate significant correlation at 𝑃 < 0.05 and 𝑃 < 0.01, respectively.


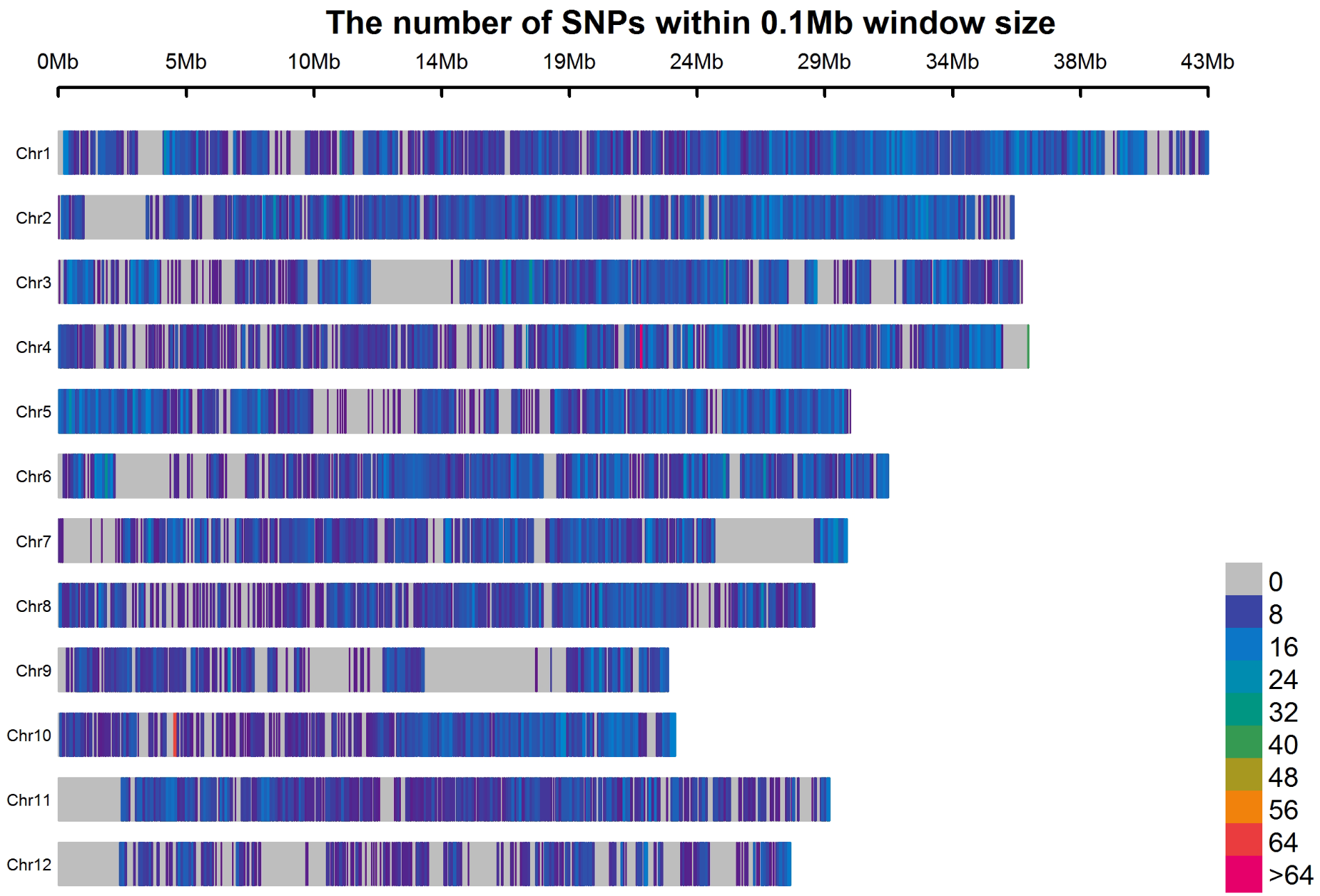


**Fig S1.** Density and chromosome distribution of polymorphic SNPs between XS09 and IR2061
